# Herbal therapy for ameliorating nonalcoholic fatty liver disease via rebuilding the intestinal microecology

**DOI:** 10.1186/s13020-021-00470-x

**Published:** 2021-07-27

**Authors:** Xiao-Fang Yang, Ming Lu, Lijiao You, Huan Gen, Lin Yuan, Tianning Tian, Chun-Yu Li, Kailiang Xu, Jie Hou, Ming Lei

**Affiliations:** 1 Critical Care Medicine, Seventh Peoples Hospital, Affiliated to Shanghai University of TCM, Shanghai, 200137 China; 2 Trauma Emergency Center, The Seventh Peoples Hospital of Shanghai University of Traditional Chinese Medicine, Shanghai, 200137 China; 3 College of Basic Medical Sciences, Dalian Medical University, Dalian, 116044 China; 4 Institute of Interdisciplinary Integrative Medicine Research, Shanghai University of Traditional Chinese Medicine, Shanghai, 201203 China

**Keywords:** Chinese herbal medicines (CHMs), Nonalcoholic fatty liver disease (NAFLD), Gut microbiota, Gut-liver axis, Intestinal microecology

## Abstract

The worldwide prevalence of nonalcoholic fatty liver disease (NAFLD) is increasing, and this metabolic disorder has been recognized as a severe threat to human health. A variety of chemical drugs have been approved for treating NAFLD, however, they always has serious side effects. Chinese herbal medicines (CHMs) have been widely used for preventing and treating a range of metabolic diseases with satisfactory safety and effective performance in clinical treatment of NAFLD. Recent studies indicated that imbanlance of the intestinal microbiota was closely associated with the occurrence and development of NAFLD, thus, the intestinal microbiota has been recognized as a promising target for treatment of NAFLD. In recent decades, a variety of CHMs have been reported to effectively prevent or treat NAFLD by modulating intestinal microbiota to further interfer the gut-liver axis. In this review, recent advances in CHMs for the treatment of NAFLD via rebuilding the intestinal microecology were systematically reviewed. The key roles of CHMs in the regulation of gut microbiota and the gut-liver axis along with their mechanisms (such as modulating intestinal permeability, reducing the inflammatory response, protecting liver cells, improving lipid metabolism, and modulating nuclear receptors), were well summarized. All the knowledge and information presented here will be very helpful for researchers to better understand the applications and mechanisms of CHMs for treatment of NAFLD.

## Introduction

Nonalcoholic fatty liver disease (NAFLD) is a clinicopathological syndrome characterized by diffuse hepatocellular globular fat without heavy alcohol consumption, and it consists of a continuum of liver conditions that vary in severity of injury and resulting fibrosis. There are two types of NAFLD: nonalcoholic simple fatty liver (NASFL) and nonalcoholic steatohepatitis (NASH) [1–3]. Epidemiological investigations have found that the prevalence of NAFLD in Asians is approximately 15% to 21%, with more than 90% of these cases accompanied by obesity, diabetes, hypertension and hyperlipidaemia [4–7].

Multiple factors contribute to NAFLD pathogenesis, as indicated in the theory of "multiple organs-multiple hits", which reveals crosstalk between the liver and multiple organs, such as the gut, white/brown adipose tissue, skeletal muscle and central nervous system [8]. During NAFLD progression, changes in both gut microbiota composition and intestinal lipid signalling can yield toxic microbiota products and induce intestinal damage, which could promote the entrance of pathogen-associated molecular proteins (PAMPs) or damage-associated molecular proteins (DAMPs) into the liver via the portal vein. Thus, the gut-liver axis, which represents the complex relationship between the liver and intestine proposed by Vannim in 2009 [9], plays an important role in NAFLD development [10].

Gut dysbiosis could shift the metabolic potential of the gut microbiota, thereby altering the bile acid metabolism of the host, which is closely linked to NAFLD pathogenesis [11]. The intestinal flora is indispensable for the conversion of bile acids and act as signalling molecules in both hepatic and extrahepatic tissues to regulate lipid and carbohydrate metabolic pathways as well as energy homeostasis [12]. That is, the intestinal flora can regulate the occurrence and development of NAFLD through bile acid metabolism and farnesoid X receptor (FXR)/ endogenous takeda G-protein-coupled receptor 5 (TGR5) signal transduction pathways, which play key roles in controlling the de novo synthesis of fat in the liver and the transport of triglycerides.

In addition, obesity and insulin resistance (IR) are associated with the development of NAFLD, and many studies have reported that the gut microbiota is also an important player in obesity [13]. In obese patients, the proportion of Firmicutes and Actinomycetes are significantly increased while that of Bacteroides is decreased, although these changes are recovered by transplanting normal flora to obese people [14, 15]. In addition, studies suggested that metabolic syndrome and insulin sensitivity could be strengthened through transplantation of intestinal flora accompanied by the recovery of gut dysbiosis [16]. Thus, the intestinal microecology and gut-liver axis, particularly the gut microbiota, likely has a significant impact on both NAFLD and its complications, such as obesity and insulin resistance. Therefore, the gut microbiota has become a novel metabolic target for the treatment of NAFLD and related metabolic disorders, and various therapeutics, including pre/probiotics, synbiotics, antibiotics, faecal microbiota transplants and herbal medicines, have been developed [17].

Recent studies have revealed that Chinese herbal medicines (CHMs) show great advantages in the treatment of NAFLD and related diseases through regulation of the intestinal microecology with desirable safety. Based on many experimental results, recent developments in NAFLD treatment with CHMs have been reviewed in this study. The relationships between NAFLD and gut microbiota were summarized, and the regulatory effects of CHMs on metabolism were discussed in detail. This review will be helpful for researchers to understand the therapeutic mechanisms of NAFLD treatment with Chinese herbal medicines (Table 1).

**Table 1:** Chinese herbal formulas exert an effect ongut mircrobiota

Chinese herbal formulas	Model	Regulatory effects on the microbiota	Key mechanisms	Ref
Gegen Qinlian decoction	Rats	Firmicutes/Bacteroidetes ratio and Oscillibacter genus↑	Improving the total amount and distribution of gut bacteria in rats	[52]
Jiangzhiligan Decoction	Rats	*Escherichia coli* and *Lactobacillus*↓	Adjusting the gut flora population of NAFLD rats and improving high-fat diet-induced NAFLD	[55]
Huatan Huoxue Recipe	Rats	Parabacteroides and Butyrivibrio Bryant and Small↑	Can partially restore the normal composition of the intestinal bacterial community	[64]
Qiwei Tiexie capsule	Rats	Modulation of gut microbiota	Protecting the liver injury in differentiated 3T3-L1 adipocytes and NAFLD by regulating the LXRα, PPARγ, and NF-κB-iNOS-NO signal pathways	[84]
Jianpi Huoxue Decoction	Rats	Regulating intestinal flora imbalance, and endotoxin production↓	Reducing the liver cell damage	[121]
Shenling Baizhu powder	Rats	Improving the abundance of intestinal microbiota, which including *Actinobacteria*, *Bacteroidetes*, *Cyanobacteria*, *Firmicutes*, *Proteobacteria*, TM7, and *Verrucomicrobia*,	Improving in intestinal permeability	[54]
Yinchenhao decoction	Rats	Regulating the diversity of the Bacteroidetes, Actinobacteria and Proteobacteria	Involving in glycerophospholipid metabolism, purine metabolism, and glutathione metabolism	[92]
Xiaozhi Decoction		Prevotella, *Bifidobacterium*, *Escherichia coli*, *Fusobacterium* and *Lactobacillus*↑	Relating to the regulation and growth of beneficial gut microbiota	[122,123]

## NAFLD and gut microbiota

**Fig. 1:**
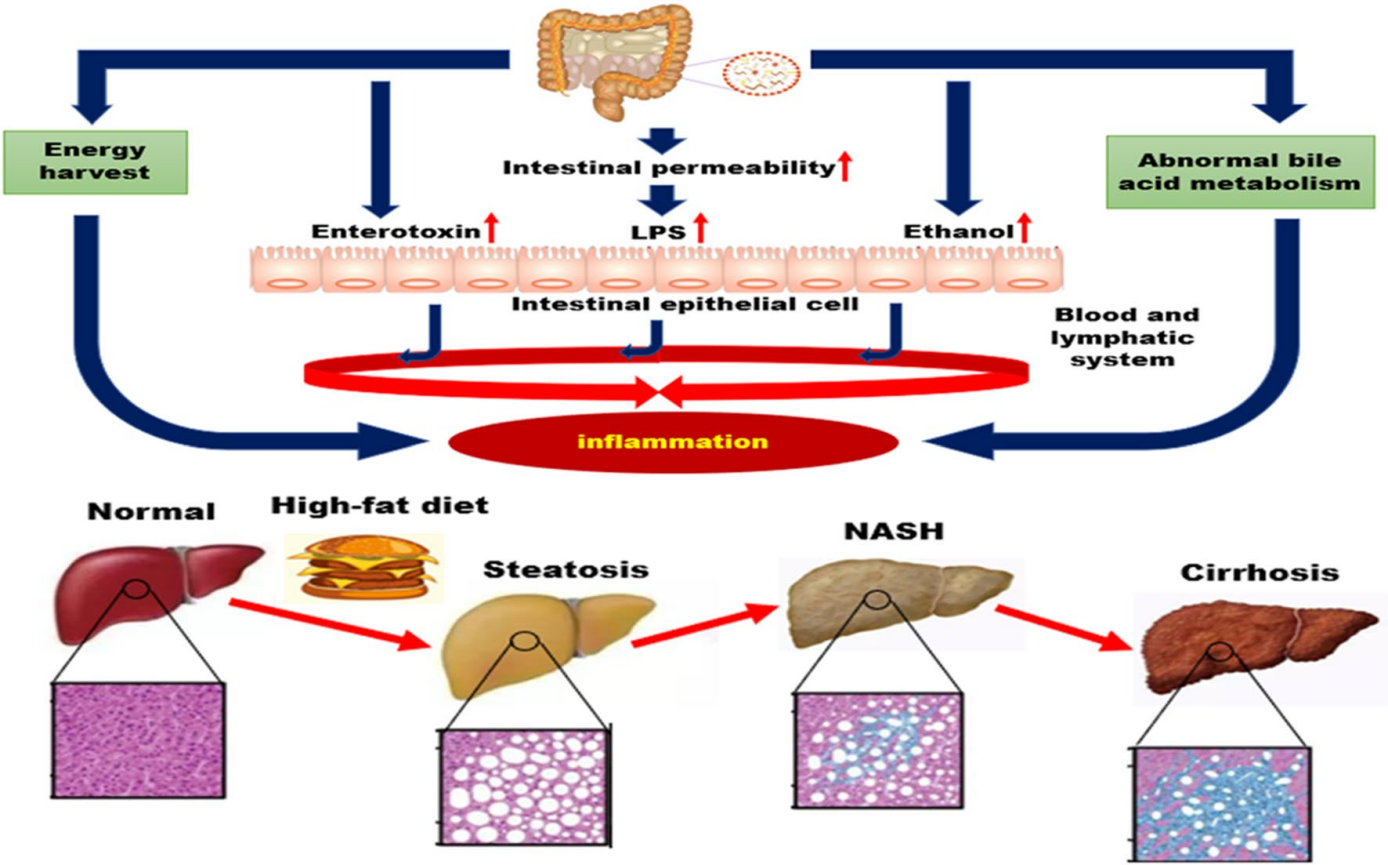
The relationships between gut microbiota and NAFLD. Enterotoxin (endotoxin, lipopolysaccharide) can damage intestinal epithelial cells and destroy their barrier function, enhancing the systemic absorption of toxins. Intestinal toxins pass from the intestinal lumen to the liver through the mesenteric circulation and lymphatic system, leading to inflammation. High fat diet rich in saturated and fatty acids can induce changes in bile acid composition of the body, thus abnormal bile acid metabolism in which bacteria live and leading to intestinal flora imbalance

The composition and quantity of gut microbiota can affect the body's metabolic functions, and the imbalance of gut microbiota will exert detrimental effects on intestinal permeability, and further induce inflammatory factors entry into the liver through blood circulation, finally result in the occurrence of NAFLD. In turn, NAFLD can further strengthen the imbalance of the gut microbiota, eventually forming a reinforcing circle [18] (Fig. 1).

### Gut microbiota imbalance can lead to the occurrence and development of NAFLD

The gut microbiota is one of the endogenous factors that promotes the pathogenesis of NAFLD [19]. Compared with healthy individuals, alterations of microbiota in NAFLD patients are usually observed at the phylum (increase in Proteobacteria), family (increase in Enterobacteriaceae and decrease in Rikenellaceae and Ruminococcaceae), and genus (increase in Escherichia, Dorea, Peptoniphilus and decrease in Anaerosporobacter, Coprococcus, Eubacterium, Faecalibacterium and Prevotella) levels [20]. Generally, the occurrence of gut dysbiosis is closely related to diet, such as a high-fat, high-fructose, and high-cholesterol diet. Animal experiments have demonstrated that the conversion of choline to methylamine could be retarded by an imbalance of intestinal microbes in mice fed a high-fat diet, thus leading to a similar progression of liver fibrosis in a choline deficiency model [21]. The deficiency of both methionine and choline can prevent phospholipid and lipoprotein synthesis, lead to abnormalities in liver lipid metabolism, and thus trigger NAFLD [22]. More importantly, once gut dysbiosis occurs, enterotoxins (endotoxin, lipopolysaccharide) can damage intestinal epithelial cells and destroy their barrier function, which subsequently enhance the systemic absorption of endotoxin. Enterotoxins passing from the intestinal lumen to the liver through the mesenteric circulation and lymphatic system can stimulate the release of various cytokines, such as tumour necrosis factor α(TNF-α), interleukin-6(IL-6), interleukin -1(IL-1), and interleukin-8(IL-8). Both acute phase proteins and transforming growth factor β (TGF-β) could form a “second strike”, resulting in simple fatty liver to NASH, liver fibrosis, and the initiation of portal hypertension [23–26] (Table 2).

**Table 2:** Herbal bioactive compounds exert an effect ongut mircrobiota

Chinese herbal formulas	Model	Regulatory effects on the microbiota	Key mechanisms	Ref
Resveratrol	Mice	*Ecoli* and Streptococcus↓, Lactobacilli and Bifidobacterium↑	Improving the gut microbiota dysbiosis and lowering both body and visceral adipose weights	[49]
Puerariae radix	Mice	PRE mediated preservation of gut barrier integrity is involved in restoring ZO-1	Decreasing intestinal permeability	[61]
Ferulic acid	Mice	The ratio of *Firmicutes* to *Bacteroidetes* and the proportions of *Firmicutes*↓, the proportions of *Bacteroidetes*↑	Altering the composition of gut microbiota	[72]
Pu-er tea extract	Mice	Bacillus, Streptococcus and Lactococcus genera ↓	Reducing inflammation, significant reducting in fatty infiltration	[66]
Curcumin	Rats	Clostridium, Bacteroides, Citrobacter, Cronobacter, Enterobacter, Enterococcus, Klebsiella, Parabacteroides, and Pseudomonas↑, Blautia and Ruminococcus↓	Ameliorating hepatic steatosis by downregulating expression of MLCK in the intestinal mucosa of rats with NAFLD, improving TJ structure of the intestinal mucosa	[67–69]
Diosgenin	Rats	*Lactobacillus murinus* and *Lactobacillus reuteri↑*	Preventing the developmentof NAFLD through the AMPK and LXR signaling pathways	[85]
Berberine	Humans	*Lactobacteriaceae*↑, *Enterobacteriaceae*↓	Improving hepatic lipid metabolism	[99]
Gynostemma pentaphyllac	Mice	*Eubacterium*, *Blautia*, *Clostridium* and *Lactobacillus*↓	Modulating he gut microbiota and suppressing hepatic miR-34a	[107]
Radix Polygoni Multiflori	Rats	The content of short-chain FA produced by gut microbial fermentationc	Reliving high contents of TG, TC in liver tissue and LPS level in portal venous	[111]
Quercetin	Mice	*Firmicutes*/*Bacteroidetes* ratio↑	Alleviation of intestinal microbiota dysbiosis, related “Gut-Liver Axis” activation	[40]
MP-A	Rats	Remodellig gut microbiota structure	Inhibiting the LPS-TLR4-NF-κB pathway activation, suppress PPAR γ and SREBP-1c expression	[95]
Green Tea Extract	Mice	Induced changes in the composition of the gut microbiota	mRNA expression levels of lipogenic and inflammatory genes were downregulated	[124]
Silymarin		*Blautia, Akkermansia, Bacteroides*↑	Attenuating hepatic steatosis	[83]
Rhizoma Coptidis	Mice	*Sporobacter termitidis*, *Alcaligenes faecalis*, *Akkermansia muciniphila*↑, *Escherichia coli*, *Desulfovibrio C21_c20*, *Parabacteroides distasonis*↓	Regulating lipid homeosta-sis by modulation gut microbiota and hepatic lipid metabolism	[86]

### NAFLD further aggravates the imbalance of the gut microbiota

Following liver dysfunction, the liver cannot dispose of intestinal-derived poisons well, which will lead to the accumulation of intestinal toxicants and damage of the intestinal mucosal barrier [27]. The absence of related antibodies, lysozymes and secretions, together with the increase in endotoxins could promote the growth of gram-negative bacteria but inhibit the growth of beneficial bacteria in the intestinal, thus the imbalance of gut microbiota will be aggravated [28–30].

As reported, forty-seven human faecal samples (25 NAFLD patients and 22 healthy subjects) were collected, and 16S rDNA sequencing was conducted by Feng Shen et al. [31]. The results showed that NAFLD patients harbored lower gut microbiota diversity than healthy subjects did. It was supposed that decreased levels of Prevotella might be adverse for adults with NAFLD, while the increased level of the genus *Blautia*, the family Lachnospiraceae, the genus *Escherichia_Shigella*, and the family *Enterobacteriaceae* may be a primary contributor to NAFLD progression. Mouzaki et al. [32] showed that patients with NASH had a lower percentage of Bacteroidetes and higher levels of *C. coccoides* than healthy subjects, suggesting that there was an opposite and diet-/BMI-independent association between the occurrence of NASH and percentage Bacteroidetes in the stool.

## CHMs for the treatment of NAFLD by rebuilding intestinal microecology

### CHMs decrease intestinal permeability by rebuilding intestinal microecology

**Fig. 2:**
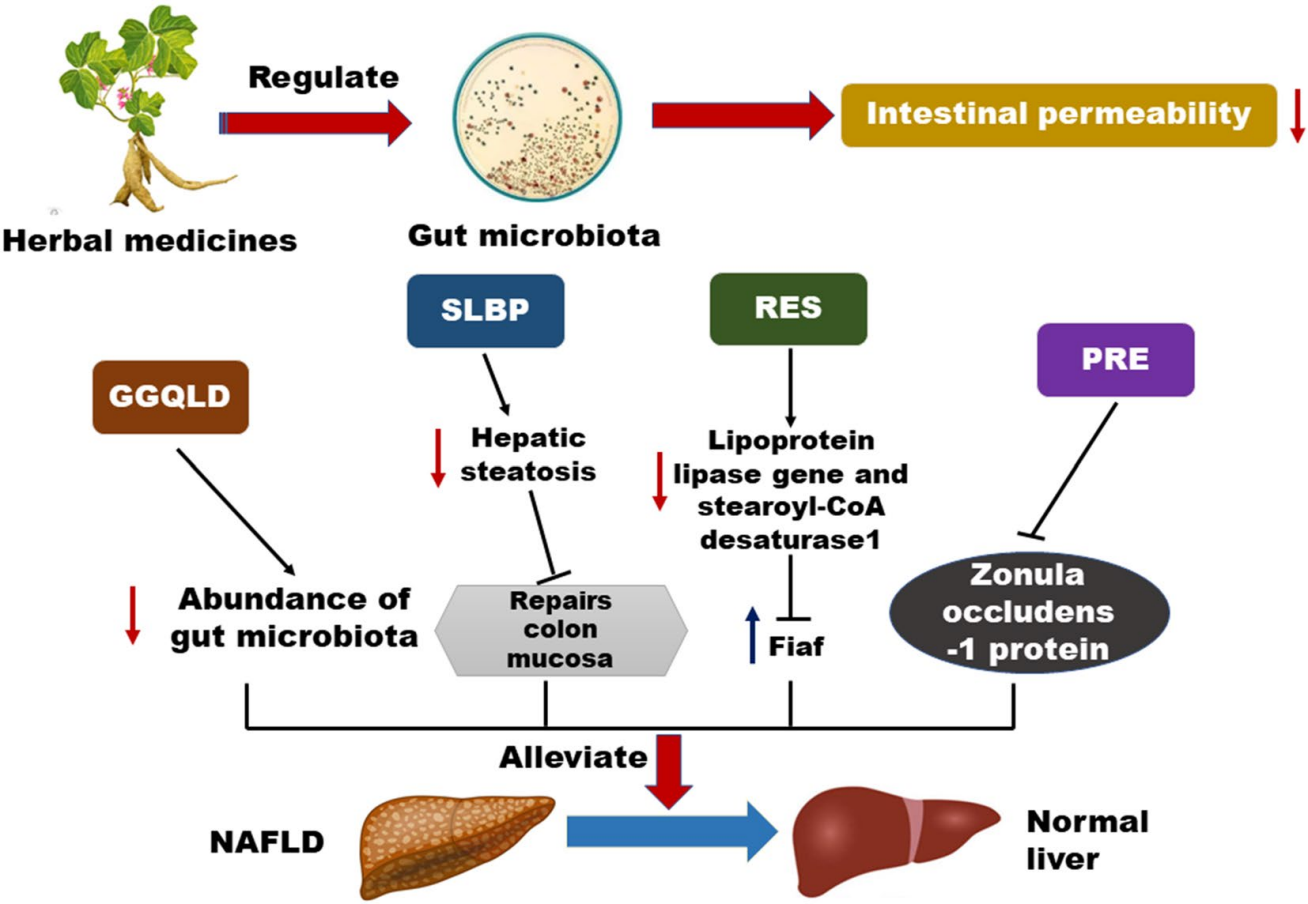
Herbal therapy for the treatment of NAFLD is linked to the gut microbiota by reducing intestinal permeability. GGQLD, RES, PRE and other factors suppress lipoprotein lipase gene and stearoyl-CoA desaturase1 in the liver, increase fasting-induced adipose factor Fiaf expression in the intestine, which regulates gut microbiota to decrease intestinal permeability

Intestinal permeability refers to the ability of the intestinal mucosal epithelium to allow various molecular substances to pass into the bloodstream in a simple and diffusion-limited way, and it is an important indicator of the function of the intestinal mucosal barrier. The relationship between intestinal mucosa and liver steatosis was initially demonstrated in studies on the mechanism of NAFLD [33]. A number of studies have shown that intestinal mucosal permeability is also significantly correlated with the occurrence and development of NAFLD [34]. Giorgio et al. [35] investigated the effects of intestinal mucosal permeability on the pathogenesis of NAFLD by the lactose-mannitol ratio method. The results showed that intestinal mucosal permeability played a crucial role in NAFLD and the level of permeability was closely related to the progression of NAFLD. Importantly, previous studies have confirmed that the permeability of the intestinal mucosa increases before morphological changes in the intestinal mucosa occur [36] and reported differences in intestinal microecology between thin and obese people. Intestinal disorders in obese people lead to the accumulation of intestinal lipopolysaccharide (LPS), resulting in increased intestinal permeability and upregulation of LPS levels in the liver via blood circulation. LPS in the liver causes fatty degeneration of liver cells and the occurrence of NAFLD. Therefore, intestinal permeability has drawn great attention in recent NAFLD studies, and Chinese herbal medicines have been reported to have a potent effect on reducing intestinal permeability [37] (Fig. 2).

Gegen Qinlian Decoction (GGQLD), a classical traditional Chinese herbal medicine, has been used to regulate lipid metabolism and attenuate inflammation during NAFLD pathogenesis [38]. Recent studies have found that GGQLD could also reduce the intestinal permeability of rats during NAFLD pathogenesis [38, 39]. In addition, the active components of GGQLD, including baicalin, glabridin, and berberine, have the ability to alleviate oxidative stress and inflammation in vivo and in vitro [39, 40]. Shenling Baizhu powder (SLBZP) is a well-known traditional Chinese medicine used in irritable bowel syndrome and non-infectious diarrhoea [41]. Y. Zhang et al. established a NAFLD rat model with a high-fat diet, and both inflammatory factors and serum biochemical indices were compared during different interventions. Serum biochemical analysis showed a decrease in serum total cholesterol (TC) levels after SLBZP treatment, which resulted in the recovery of liver function. Additionally, SLBZP reduced the serum levels of endotoxin, TNF-α, and interleukin-1β (IL-β) and the expression of TLR4 pathway-related proteins. Pathological examination showed that SLBZP alleviated hepatic steatosis and repaired the colonic mucosa. Microbiome analysis revealed that SLBZP improved the abundance of intestinal microbiota, including Actinobacteria, Bacteroidetes, Cyanobacteria, Firmicutes, Proteobacteria, TM7, and Verrucomicrobia, and decreased the levels of LPS in the portal vein, which may be related to the improvement in intestinal permeability [42]. Jiangzhi Ligan Decoction, composed of alisma, cassia seed, *Salvia miltiorrhiza*, turmeric, seaweed and lotus leaf, has been found to be effective in the treatment of NAFLD and promotes protection of the liver. Jiangzhi Ligan Decoction has been reported to inhibit the excessive growth of *Escherichia coli* and promote lactic acid bacteria in NAFLD, which is beneficial for reducing intestinal permeability and thus preventing the progression of NAFLD [43].

In addition to herbal recipes, active compounds in herbal medicines have also been studied. Resveratrol (3,5,40-trihydroxy-trans-stilbene, RES) is a natural polyphenolic compound found in grapes that has cardioprotective, anti-inflammatory, antioxidant, and antitumour properties. Recently, resveratrol has been reported to have anti-obesity effects [44], and it could regulate the composition of the gut microbiota, which systemically increased the expression of the Fiaf gene in the gut and inhibits the expression of the fatty acid biosynthesis gene stearoyl-CoA desaturase 1 and lipoprotein lipase gene in the liver. Resveratrol also inhibited the expression of lipogenic genes (Ppar-g, Acc1, and Fas) or adipogenic genes in visceral adipose tissue. These results indicated that resveratrol significantly reduced visceral fat weight, lipid levels and blood glucose in HFD mice. Resveratrol modulated HFD-induced dysbiosis of the gut microbiota, increased the ratio of Bacteroidetes to Firmicutes, and decreased the Enterococcus faecalis amount and intestinal permeability. In addition, resveratrol significantly upregulated the expression of fasting-induced adipokines (Fiaf, a key gene negatively regulated by gut microbes) in the intestine.

Puerariae radix (PRE) has abundant isoflavones, including daidzin, daidzein and puerarin [45], and it has been used for the treatment of acute dysentery, pain, diabetes, measles, fever or diarrhoea [46]. In addition, medications based on Puerariae radix have been found to be useful in the treatment of alcohol-related problems by acting as an anti-intoxication and anti-drinking agent [47, 48]. The beneficial effects of PRE on NAFLD have been reported to be associated with a reduction in intestinal permeability [49]. Additionally, PRE could preserve intestinal barrier integrity by upregulating the level of the intestinal tight junction protein zonula occludens-1. These findings suggested a mechanism for the improvement of NAFLD by Puerariae radix.

### CHMs reduce inflammation by regulating gut microbiota

**Fig. 3:**
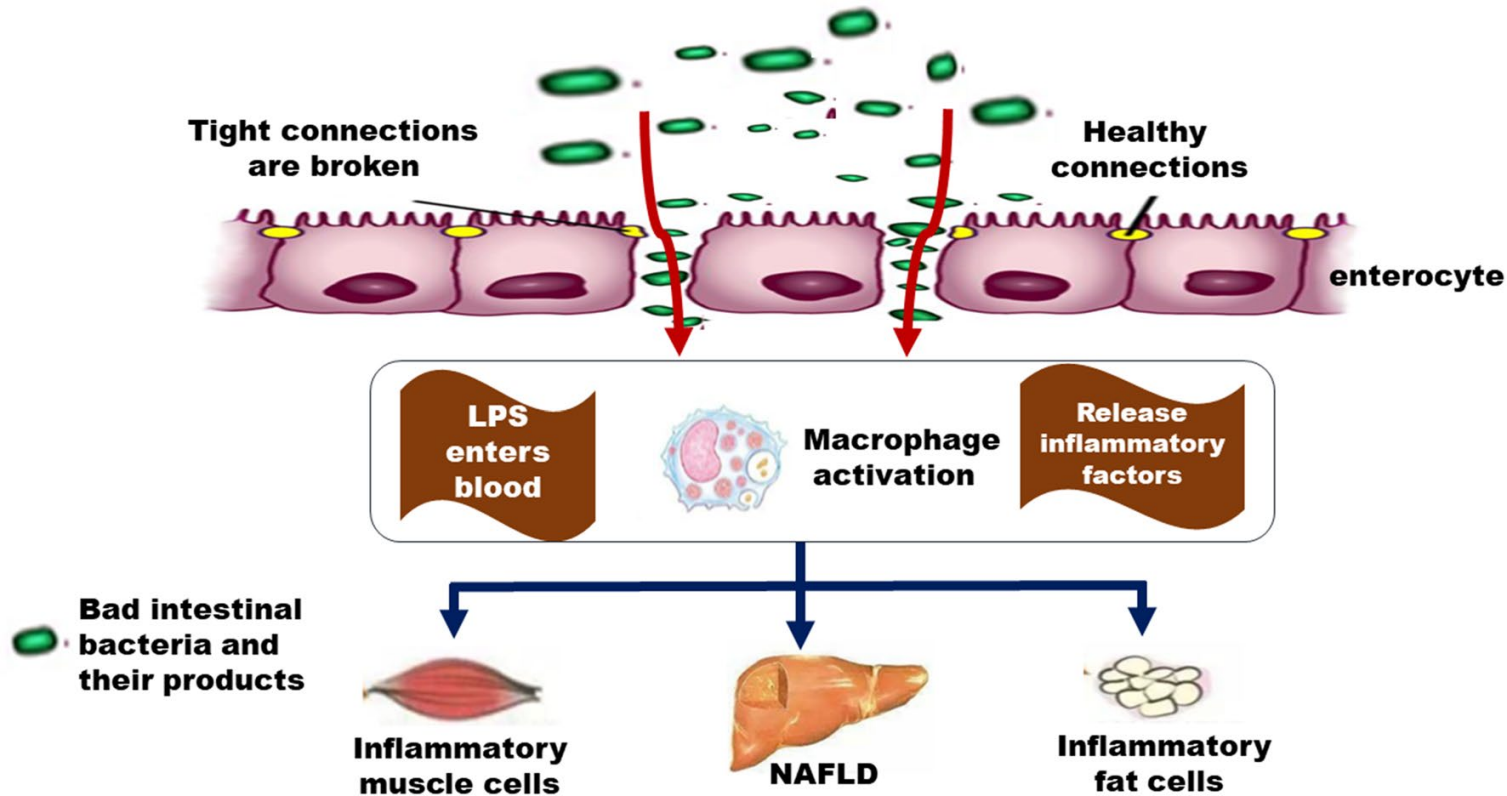
The relationships between inflammation and NAFLD. Harmful gut bacteria release toxins that enter the circulatory system, which in turn, activating macrophages and releasing inflammatory cytokines, leading to inflammation of fat and muscle cells, and aggravating NAFLD

As the “multiple hit model” or “two hit model” illustrates, an imbalance in intrahepatic lipid metabolism induced by insulin resistance (IR) is considered the first hit [50]. The insulin receptor activates the downstream MAPK pathway and related nuclear transcription factors (such as NF-κB) through autophosphorylation and then immediately releases inflammatory factors and reactive oxygen species, leading to an intracellular inflammatory response and oxidative stress and finally exacerbating the development of NAFLD. In addition, activated insulin receptors could stimulate hepatic stellate cells to release profibrotic factors, promote collagen formation, and ultimately cause fibrosis of the liver. These intrahepatic pathological changes initiated by insulin resistance are collectively referred to as the second strike or multiple strikes. In these attacks, the inflammatory response can cause the development of steatohepatitis and further promote the process of oxidative stress and fibrosis. Therefore, inhibition of inflammation in the treatment of NAFLD is critical (Fig. 3).

Huatan Huoxue Recipe could strengthen the spleen and protect the liver, remove dampness, promote blood circulation and prevent blood stasis. Recent clinical studies have shown that the Huatan Huoxue Recipe can relieve insulin resistance in NAFLD patients by resolving phlegm, promoting blood circulation [51], significantly reducing the levels of ALT and TG, and lowering the BMI and CT value of the liver/spleen. WX Zhao et al. [52] showed that Huatan Huoxue Recipe could effectively improve liver function by upregulating the levels of ADPN, AdipoR2 and p-AKT (Ser473) in liver tissue and downregulating p-NF-κBp65 (Ser536), conversely lowering the levels of blood lipids and GLU, thus resulting in a reduction in inflammation and the amount of fatty tissue in the liver of NAFLD. Furthermore, the Huatan Huoxue Recipe could have a two-way therapeutic effect on NAFLD by increasing the number of probiotics, such as Christensenella, Bifidobacterium, and Psychrobacter, and reducing the number of Staphylococcus and Streptococcus, which produce pathogenic enterotoxins, damage the permeability of the intestinal mucosa, and cause an imbalance in the intestinal microecology of the body. It is suggested that the mechanism of the Huatan Huoxue Recipe in the treatment of NAFLD may be related to the inhibition of the number of pathogenic bacteria and the inflammatory response.

Pu'er ripe tea (PTE) is a fully fermented tea produced by a special process in Yunnan large-leafed species that has various biological effects, such as antibacterial effects [53]. A recent study used C57BL/6 N mice as an experimental animal model. NAFLD was induced by a HFD, and then PTE was administered through drinking water. The results showed that PTE could modulate the intestinal flora imbalance, increase the levels of Akkermansia and significantly decrease levels of thick-walled phyla, which are significant guides in the development of NAFLD, were detected by fluorescence real-time quantitative PCR. The results also revealed that the expression of inflammatory factors TNF-α, IL-1β and lipid metabolism-related genes FAS and SREBP-1c were significantly decreased in the NAFLD + 0.4% PTE group compared with the NAFLD group. PTE can also significantly reduce inflammatory markers and lower the NAFLD index, which indicated that it has the potential to prevent NAFLD [54].

As an active compound, curcumin usually acts as an antioxidant and immunomodulator by scavenging free radicals [55]. Hou H. T et al. [56] established a NAFLD rat model by a HFD and studied the effects of curcumin on secretory immunoglobulin A (sIgA) and oxidative stress. The results showed that curcumin could improve the intestinal viscosity and oxidative stress status by increasing intestinal SIgA levels, thereby reducing the inflammatory response. Similarly, in another study [57], after curcumin intervention, the expression of myosin light chain kinase (MLCK) in the small intestinal mucosa was significantly reduced, the tight junction structure was restored, the intestinal mucosal permeability was reduced, and the proportion of thick-walled phyla was decreased, in which the proportion of bacilli and the ratio of beneficial bacteria Lactobacillus fermentum were increased. Furthermore, the levels of the inflammation-related factors ALT, AST, LPS and DAO were also significantly reduced. The above results indicated that curcumin may improve the tight junction structure of the intestinal mucosa and reduce the inflammatory response by downregulating the expression of MLCK in the intestinal mucosa.

Ferulic acid (FA) is a phenolic acid that is widely found in plants. It binds to and incorporates polysaccharides and proteins in the cell wall. It has received attention for its hepatoprotective, antimicrobial, anti-inflammatory, antioxidant, antitumour, and immunomodulatory properties [58, 59]. In addition, FA produced by Lactobacillus fermentans affects developmental growth through a dTOR-mediated mechanism. FA has been shown to alleviate nonalcoholic fatty liver disease [60]. In addition, studies have shown that FA supplementation alters the composition of the gut microbiota, particularly by modulating the ratio of Firmicutes to Bacteroidetes and decreasing the generation of indole-3-acetic acid [1]; however, in vitro, FA (25 and 50 μg/mL) treatment significantly reduces cellular lipid accumulation with no obvious cytotoxicity, which is partially mediated by the suppression of ERK1/2, JNK1/2/3, and HGMB1 expression [61]. Moreover, at the cellular level, FA and the trans and cis isomers of FEF77 were able to protect human endothelial cord vein (HECV) cells from the oxidative damage and inflammatory response induced by exposure to hydrogen peroxide, as measured by cell viability and ROS production assays [62]. FA has the potential to ameliorate NAFLD, which may be related to the regulation of specific gut microbiota and genes related to TG and TC metabolism [1].

### CHMs ameliorate NAFLD by targeting intestinal nuclear receptors

**Fig. 4:**
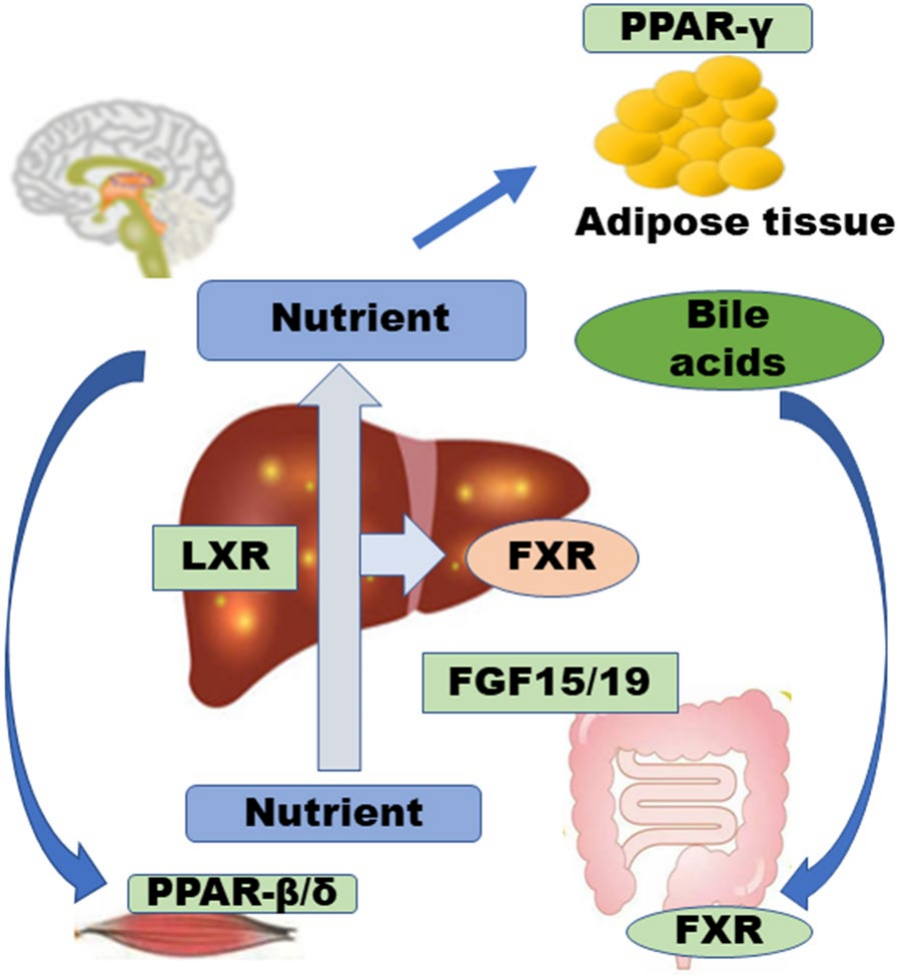
In the fed state energy flux is reversed and FXR, LXR, PPARβ/δ and PPARγ are mainly involved in nutrient absorption from the gut and distribution from gut/liver to peripheral tissues, such as adipose tissue and muscle. After meals, bile acids activate intestinal FXR, promoting nutrient absorption and maintaining a barrier to the gut microbiome. Absorbed dietary lipids are transported into the circulation as chylomicrons. Hepatic FXR promotes post-prandial TAG-rich lipoprotein clearance. Excess cholesterol is removed from the body by reverse cholesterol transport under the control of the FXR-stimulated enterokine FGF19 (FGF15 in rodents) and/or activation of hepatic LXR by oxysterols. FGF15/19 attenuates post-prandial hyperglycemia by enhancing hepatic glycogenesis

Nuclear receptors (NRs) are ligand-activated transcription factors that regulate several important metabolic processes, including liver lipid metabolism, glucose metabolism, and bile acid metabolism. The maladjustment of these processes contributes to the onset and development of NAFLD [63]. Therefore, NRs have emerged at the forefront of novel treatments for NAFLD. Some NRs are already pharmacologically targeted in metabolic disorders, such as hyperlipidaemia (peroxisomal proliferator-activated receptor α [PPARα], fibrates) and diabetes (PPARγ, glitazones), and have potential applications for NAFLD [64]. Other NRs, including vitamin D receptor (VDR), constitutive androstane receptor (CAR), pregnane X receptor (PXR), nuclear bile acid receptor FXR and RAR-related orphan receptor γ2 (RORγt), are also being research. Thus, the development of combined ligands for NR isoforms, such as PPARα/δ ligands, has aroused attention [65–67]. Because NAFLD is a metabolic syndrome associated with insulin resistance (IR) of the liver, NRs involved in lipid and glucose metabolism and energy balance are very attractive therapeutic targets in NAFLD treatment [68]. Moreover, NRs may also be important for NAFLD-related diseases, such as cardiovascular disease (Fig. 4).

FXR is both a nuclear receptor for bile acids and an inhibitor of the de novo formation of bile acids in the liver, and it is highly expressed in hepatocytes and bile duct epithelial cells. FXR is involved in cholesterol metabolism, glycerol triglyceride metabolism, glucose metabolism, and energy consumption, especially in the maintenance of normal lipid metabolism and control of the inflammatory response; therefore, FXR deficiency increases hepatocyte triglyceride levels and contributes to the development of NAFLD. The loss of FXR is clinically characterized by fatty degeneration, inflammatory infiltration and fibrillation of the liver [69]. Dekaney et al. [70] found that after ileocecal resection of FXR-deficient germ-free mice, the mice showed a weakened FXR-mediated Wnt/β-catenin signal and failed to induce bile acid conversion compared with wild-type mice. In a study of 113 patients with NAFLD, Bechmann et al. [71] found that CYP7A1 and bile acid transporter sodium ion/taurocholate isotransfer (Na + /taurocholate cotransporter, NTCP) were increased with elevated levels of free fatty acids in the body, thereby leading to fatty hepatitis. The increased expression level of NTCP indicated that the rapidity of bile acid synthesis and the concentration of bile acids in plasma were associated with NAFLD severity. The application of the FXR ligand WAY-362450 to NASH mice reduced liver inflammation and fibrillation, and the application of WAY-362450 downregulated the expression of transforming growth factor-β1 and matrix metalloprotein and the concentration of bile acids in plasma. The expression of genes related to hepatic fibrillation, such as matrix metallopeptidase 2 and tissue inhibitor of metalloproteinases 1, decreases serum alanine transaminase and aspartate aminotransferase levels and reduces keratinocyte derivatization chemokines and monocytes. The production of inflammatory factors, such as chemotactic protein 1, is useful in the treatment of NAFLD.

Bile acid levels in humans are positively correlated with serum triglyceride levels; therefore, high bile acid levels are associated with the development of NAFLD. During reabsorption in the ileum, bile acids are simultaneously secreted by intestinal cells into the hepatic circulation as fibroblast growth factor 19 (FGF19), which is associated with the fibroblast growth factor receptor 4 complex, activates the two major signalling pathways c-Jun N-terminal kinase (JNK) and extracellular signal-regulated kinase (ERK), and inhibits the transcription of the cholesterol 7a-hydroxylase (CYP7A1) gene, which is a rate-limiting enzyme in the bile acid synthesis reaction, thereby inhibiting the synthesis of bile acids. The level of FGF19 in the serum of NAFLD patients was reduced, which further revealed the presence of bile acids, FGF19 and NAFLD. The clinical significance of FGF19 varies in different age groups. Reports have indicated that the level of FGF19 in adults with NAFLD cannot be used to assess liver histological changes alone [72], although the level of FGF19 in children with NAFLD is associated with the degree of hepatic lipid metabolism and fibrillation [72]. In summary, FXR egulates bile acid (BA) synthesis, transport, and hepatic intestinal circulation by modulating the expression of related genes in the liver and small intestine. The composition of the intestinal flora is associated with metabolic diseases, particularly obesity and nonalcoholic fatty acid disease (NAFLD).

Silymarin is extracted from the milk thistle plant (Silybum marianum) and has been used for centuries as a natural treatment for hepatobiliary diseases. Considering the therapeutic potential for liver disease, Ni et al. [60] investigated the efficacy of silymarin on hepatic steatosis and predicted possible effects on lipid metabolic pathways in a mouse model of HFD-induced nonalcoholic fatty liver disease. The results indicated that silymarin could attenuate hepatic steatosis, as measured by oil red O staining and TG levels. Furthermore, compared with INT-747, a potent and selective FXR agonist, silymarin preserved plasma high-density lipoprotein cholesterol (HDL-C) and reduced low-density lipoprotein cholesterol (LDL-C). Furthermore, Li et al. reported that silymarin protects against diet-induced obesity and nonalcoholic fatty liver disease by modulating the composition of the gut microbiota. Compared with the HFD group, mice in the silymarin-treated group had significantly lower ratios of Firmicutes to Bacteroidetes, Firmicutes, Lachnoclostridium, Mollicutes_RF9 and Lachnospiraceae_UCG-006, which were reported to be potentially related to diet-induced obesity, and increased levels of Blautia, Akkermansia, and Bacteroides, which are known to have beneficial effects on improving NAFLD. Silymarin also showed a facilitating effect on well-known beneficial bacteria, such as Lactobacillus and Alloprevotella [73].

Qiwei Tiexie capsule (QWTX) is a representative prescription of Tibetan medicine that is widely used for the long-term treatment of NAFLD and chronic liver disease. A study by Suolang et al. found that QWTX could decrease lipid accumulation without cell cytotoxicity in 3T3-L1 preadipocyte cells. In NAFLD, QWTX attenuated liver steatosis, fat vacuoles and inflammation according to HE staining and electron micrography. In terms of oxidative stress biomarkers, QWTX treatment resulted in decreased serum FFA levels and increased serum NO levels. In liver tissues, SOD levels and MDA levels returned to normal after QWTX treatment. QWTX also downregulated NF-κB and CYP2E1 compared to NAFLD. In addition, downregulation of LXRα, PPARγ and iNOS was also observed by QWTX in 3T3-L1 adipocytes and in NAFLD animal models [74].

Diosgenin, which is abundant in Rhizoma Dioscoreae nipponicae, has been shown to lower high plasma glucose levels and improve the distorted tissue lipid profile in HFD-streptozotocin-induced diabetic rats [75]. Cheng et al.’s study showed that diosgenin inhibits LXRα and activates the AMPK pathway, thereby reducing hepatic lipid accumulation [76]. SREBP1 is also a major target of LXRα, which inhibits HG-induced upregulation of SREBP-1c mRNA, upregulates lipogenesis and can be partially blocked by AMPK inhibitors, suggesting that other pathways may also be involved in the lipid-lowering effects of diosgenin. Diosgenin also ameliorates HFD-induced liver dysfunction, and these data suggest that diosgenin is a potential agent for the prevention of NAFLD via the AMPK and LXRα pathways.

Rhizoma Coptidis (RC) is a widely used traditional Chinese medicine that has a significant lipid-lowering effect. RC can be used to clear heat and purge fire, resolve phlegm to activate meridians, promote blood circulation to remove blood stasis, remove dampness and nourish the kidney and intestine. A study evaluated the lipid-lowering mechanism of high doses of xanthophyll alkaloids (daily dose 140 mg/kg for 35 days). High lipid and high cholesterol induced hyperlipidaemia in B6 mice. After treatment, the serum lipid parameters were determined to assess the expression of lipid metabolism-related genes and the pathways of sterol regulatory element binding proteins (SREBPs) and bile acid signalling in mice. Additionally, Illumina sequencing was performed to study the differences in the intestinal microflora of B6 mice. The results showed that RC alkaloid feeding significantly enhanced the abundance of Sporobacter termitidis, Alcaligenes faecalis, and Akkermansia muciniphila in the gut of mice but decreased the abundance of Escherichia coli, Desulfovibrio C21_c20, and Parabacteroides distasonis [77].

Berberine (BBR) was also found to inhibit bile salt hydrolase activity in the intestinal flora, significantly increasing the intestinal bile acids bound to bovine scalp, especially bovine sulfocholic acid (BSH). Both BBR and taurocholic acid treatment activated the intestinal FXR pathway and decreased the expression of the fatty acid transporter CD36 in the liver. These results suggest that the lipid-lowering effects of BBR may act primarily through the regulation of bile acid metabolism and the subsequent ileal FXR signalling pathway in the gut. Taken together, they provide evidence for a novel mechanism of action of BBR in the gut that includes inhibition of BSH, elevation of taurocholic acid and activation of FXR, which inhibit hepatic expression of CD36 and subsequently reduce the uptake of long-chain fatty acids in the liver [78].

### CHMs improve lipid metabolism by regulating gut microbiota

**Fig. 5:**
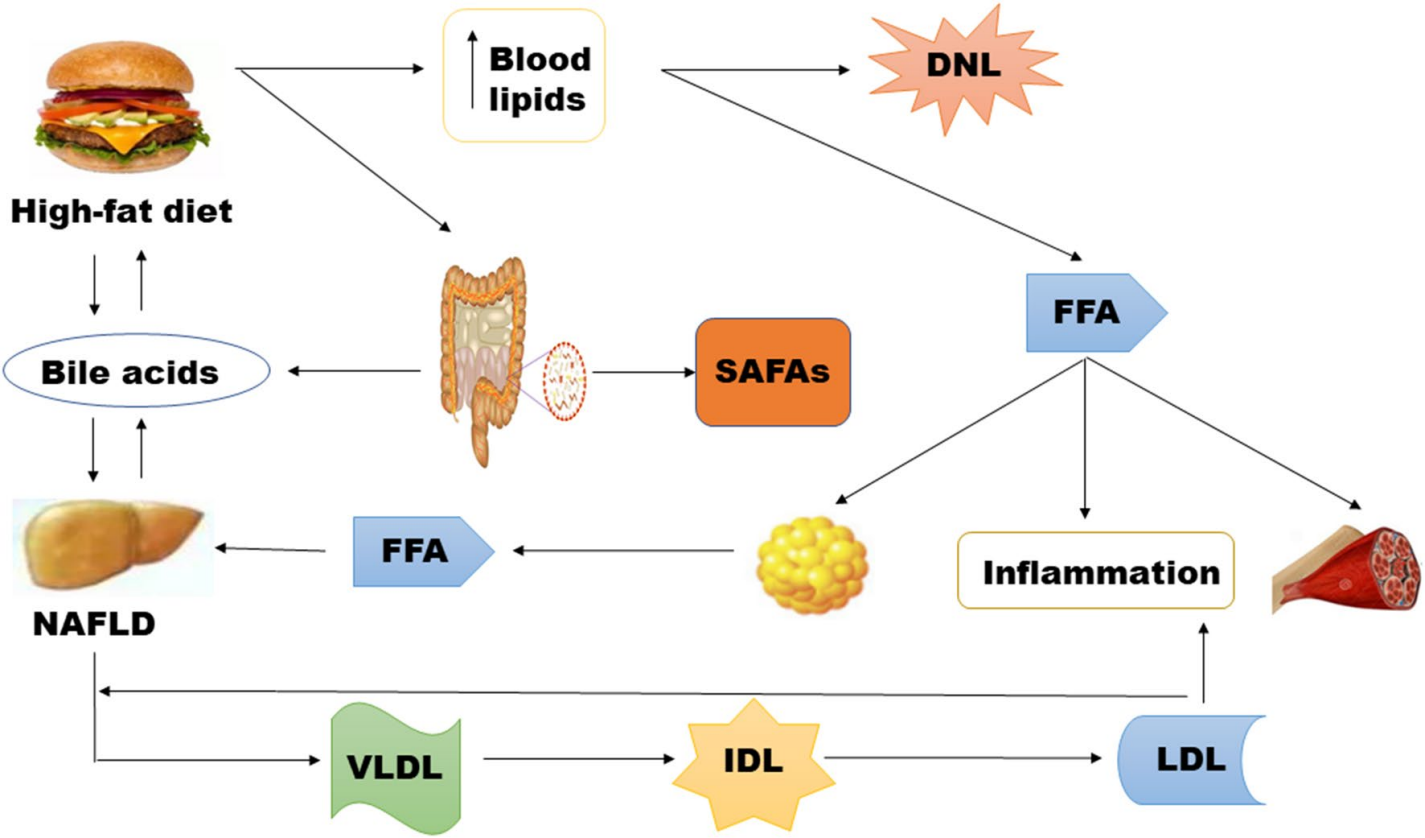
The relationships between lipid metabolism and NAFLD. Foods with high fat and sugar can cause elevated blood lipid levels, promote the process of DNL, and increase the synthesis of FFA that further accumulates in the liver. Adipose tissue dysfunction and inflammation also play important roles in the pathological development of NAFLD. *FFA* free fatty acid, *VLDL* very low density lipoprotein, *IDL* intermediate-density lipoprotein, *LDL* low-density lipoprotein, *SCFAs* short-chain fatty acids

The liver is the main organ that regulates lipids in the body. A high-fat and high-sugar diet can elevate blood lipid levels and enhance de novo lipogenesis (DNL), thereby leading to increases in the synthesis of free fatty acids (FFAs), which further accumulate in the liver. When the rate of lipid accumulation in the liver exceeds its catabolic rate, triglycerides (TGs) will accumulate in liver cells, resulting in the formation of lipid droplets and inflammation, which is one of the hallmarks of liver fibrosis, nonalcoholic fatty liver, cirrhosis and liver cancer [79]. Adipose tissue dysfunction and inflammation also play an important role in the pathological development of NAFLD (Fig. 5). Recent studies have found that intestinal flora dysregulation also played an important role in regulating metabolic and inflammatory pathways in the liver. A variety of bacterial metabolites, such as short-chain fatty acids, lipopolysaccharides, endotoxins, etc., are absorbed in the intestinal tract, which induce inflammation and pathological changes in the liver [80, 81]. In the process of liver lipid metabolism, the imbalance of lipid supply and metabolism is the main reason for NAFLD.

The original recipe for Yinchenhao Decoction was obtained from Treatise on Cold Damage Diseases. Modern studies have found that it has a wide range of pharmacological effects, and it is often used in the clinical treatment of NAFLD and other related diseases [82, 83]. It has been reported that Yinchenhao Decoction can regulate the diversity of Bacteroidetes, Actinobacteria and Proteobacteria in NAFLD model rats, in which the abundance of 16 species was significantly recovered. Thirteen treatment-related liver metabolites were identified, and the three strongest metabolic pathways involved were glycerophospholipid metabolism, purine metabolism, and glutathione metabolism [84].

The thick-shelled mussel *Mytilus coruscus* is a marine mussel species mainly distributed in the Korean Peninsula, Japanese Hokkaido and Chinese Yellow Sea coastal areas [59]. Wu et al. [85] isolated a new mussel polysaccharide, α-D-glucan (MP-A), from M. coruscus. The results showed that MP-A supplementation reduced lipid levels, intrahepatic lipid accumulation and NAFLD activity scores in HFD-fed rats. Furthermore, 16S rDNA sequencing analysis of gut microbiota samples revealed that a HFD could induce microbial dysbiosis. However, MP-A supplementation remodelled the gut microbiota composition and inhibited LPS-TLR4-NF-κB pathway activation and subsequent inflammatory factor secretion. Additionally, MP-A regulates lipid metabolism by promoting the production of short-chain fatty acids and inhibiting the expression of PPARγ and SREBP-1c. These results imply that MP-A could inhibit NAFLD by modulating the gut microbiota and associated enterohepatic axis signalling pathways and act as an oral supplement to protect the liver.

Berberine is an alkaloid isolated from a variety of medicinal plants, such as *Coptis chinensis* and *Berberis vulgaris*. BBR has been used as an over-the-counter (OTC) drug for the treatment of bacterial diarrhoea in China, and its safety in humans has been well established [86, 87]. We have reported that berberine was effective in the treatment of type 2 diabetes and hyperlipidaemia [88, 89]. In a recent study, berberine supplementation was found to significantly reduce serum and liver lipid contents in high-fat diet-fed rats. In addition, the level of SOD was significantly increased while the level of MDA was decreased in the livers of rats. Oil red O and HE staining results showed that liver steatosis was ameliorated in the berberine-supplemented group. In addition, berberine induced an increase in SIRT1 expression but a decrease in UCP2 expression. The regulation of the hepatic SIRT1-UCP2 pathway may be an important mechanism by which berberine exerted beneficial effects on NAFLD rats [90].

*Gynostemma pentaphylla* (GP), a trailing plant belonging to the Cucurbitaceae family, has been widely used either alone or as a principal component in herbal formulae for the prevention of hyperlipidaemia and hyperglycaemia in Asian countries. Pharmacological studies have revealed that GPs possess various bioactivities, including antioxidative [91], anti-inflammatory [92], hypoglycaemic [93, 94], lipid-lowering [95], and hepatoprotective effects [96]. Jia N et al. [97] evaluated the role of GP in the treatment of NAFLD in vivo and found that GP was effective in reducing lipid metabolism and protecting hepatocytes, likely by modulating gut microbiota and suppressing miR-34a.

Short-chain fatty acids (SCFAs), mainly acetic acid, propionic acid, isobutyric acid, butyric acid, isovaleric acid and valeric acid, are important metabolites of intestinal flora rather than the host [98], among which the proportion of acetic acid, propionic acid and butyric acid is as high as 85% [99]. SCFAs not only provide energy to intestinal mucosal cells but also promote cell metabolism and growth, regulate intestinal pH, and prevent intestinal dysfunction [100]. The extract of Radix polygonum multiflorum and 2,3,5,4'-tetrahydrocysteine-2-O-β-d- glucopyranoside (TSG) was reported to reduce total short-chain fatty acid (SCFA) levels in the intestines of rats on a HFD with sex differences. Radix polygonum multiflorum and TSG significantly reduced the levels of butyric acid, propionic acid, and acetic acid in the intestinal tract of male rats fed a high-fat diet but reduced the hepatic lipid content and endotoxin levels in all experimental animals. Radix polygonum multiflorum decreased the intestinal propionic acid content as well as the hepatic lipid content in female rats fed a high-fat diet. Low doses of TSG increased the acetic acid content and lipid and endotoxin levels [101].

### CHMs reduce liver cell damage by modulating gut microbiota

Hepatocyte injury is a complex pathological process induced by many factors that results in apoptosis or necrosis of liver cells. In terms of its pathogenesis, both hyperlipidaemia and insulin resistance are two risk factors for NAFLD, and a series of metabolic disorders triggered by hyperlipidaemia lead to the formation of NAFLD. Under normal conditions, the liver lipid content accounts for 2–4% of the liver wet mass, whereas once the liver fat content exceeds 5% of the liver wet mass, a fatty liver can be diagnosed. Studies have shown that TG and TC in the blood of NAFLD patients are significantly higher than those without fatty liver [102].

Jianpi Huoxue Recipe is composed of Salvia miltiorrhiza, atractylodes, turmeric, alisma and other components. Herbal medicines in this prescription have anti-obesity and liver protection functions; moreover, the active ingredients in both Salvia miltiorrhiza and turmeric revealed scavenging activities and inhibition against lipid peroxidation [103–105]. Studies have disclosed that the positive liver-protective effects of Jianpi Huoxue Decoction are closely related to its ability to regulate intestinal flora imbalance and reduce endotoxin production [106, 107]. Jianpi Huoxue Decoction treatment alleviated pathological damage to liver tissue and decreased the content of TG in the liver, activity of serum ALT and content of TNF-α in portal plasma. CD68 immunohistochemical staining of liver Kupffer cells showed that the positive CD68 staining of rats in the Jianpi Huoxue Decoction group was significantly less than that of the model group [108].

Xiaozhi Decoction (Patent No. China, 201110416602. 1) can significantly improve insulin resistance and oxidative stress in NAFLD and can also reduce liver fat accumulation and inflammatory reactions [109]. Zhu, Q. et al. [110] established a NAFLD mouse model using a 60% high-fat diet. After successful modelling, the expression levels of tumour necrosis factor-alpha, interleukin-6 and lipopolysaccharide were detected by enzyme-linked immunosorbent assay. Two liver tissues were collected for paraffin embedding, haematoxylin-eosin staining and oil red O staining. In the Xiaozhi Decoction group, the bacterial counts of Prevotella, Bifidobacterium, Fusobacterium and Lactobacillus were much higher in the high-dose group and middle-dose group than in the model group. However, the bacterial count of *Escherichia coli* was lower than that of the model group. The results showed that Xiaozhi Decoction can improve IR and liver function and reduce liver fat accumulation and the inflammatory response, which may be related to the regulation and growth of beneficial gut microbiota.

Fermented green tea (FGT) is a novel fermented product of dried green tea leaves fermented by *Bacillus subtilis*, a microorganism used to produce fermented soybean products in East Asia, particularly in Korea. Recent studies have demonstrated that both green tea and its processed products exert beneficial effects on lipid metabolism. SEO et al. [111] reported that FGT induced changes in the composition of the gut microbiota in obese mice (e.g., the Firmicutes/Bacteroidetes and Bacteroides/Prevotella ratios), which may be the mechanism for fat and body weight loss and reductions of liver cell injury and fatty liver symptoms.

Quercetin is derived from the skins and leaves of *Quercus iberica* and the leaves of *Apocynum lancifolium* Rus. It is a good expectorant and cough suppressant and has some asthmatic effects. David Porras et al. [40] used C57BL/6J mice fed a high-fat diet (HFD) supplemented with or without quercetin for 16 weeks. The microbiota composition was determined via 16S ribosomal RNA Illumina next-generation sequencing, and metagenomic studies revealed HFD-dependent differences at the phylum, class and genus levels that led to dysbiosis, which was characterized by an increase in the Firmicutes/Bacteroidetes ratio and gram-negative bacteria and a dramatic increase in the detection of the *Helicobacter* genus. These results indicated that quercetin could counteract lipid metabolism gene expression deregulation, alleviate intestinal microbiota dysbiosis and reduce liver damage, which was related to “gut-liver axis” activation.

## Conclusions and perspectives

In recent decades, herbal therapy has been validated as an effective therapeutic strategy for the treatment of NAFLD and its related disorders [22, 111–113]. The concurrence and development of NAFLD had closed relationship with the imbalance of intestinal microecology. CHMs could recover the dysbiosis of intestinal microecology in NAFLD *via* different mechanisms, such as decreasing intestinal permeability, reducing the inflammatory response, protecting liver cells, improving lipid metabolism, and adjusting nuclear receptors. To better understand the efficacy of CHMs for the treatment of NAFLD *via* modulation of gut microbiota, researchers have attempted to depict the potential material basis of CHMs and their key roles as modulators to maintain the intestinal microbiota. In the future, more state-of-the-art techniques including microbial metagenome, microbial metabolomics, microbial proteomics and multiomics integration analysis should be applied to discover the key targets and the molecular mechanisms of herbal constituents in the management of NAFLD [114, 115].

**Fig. 6:**
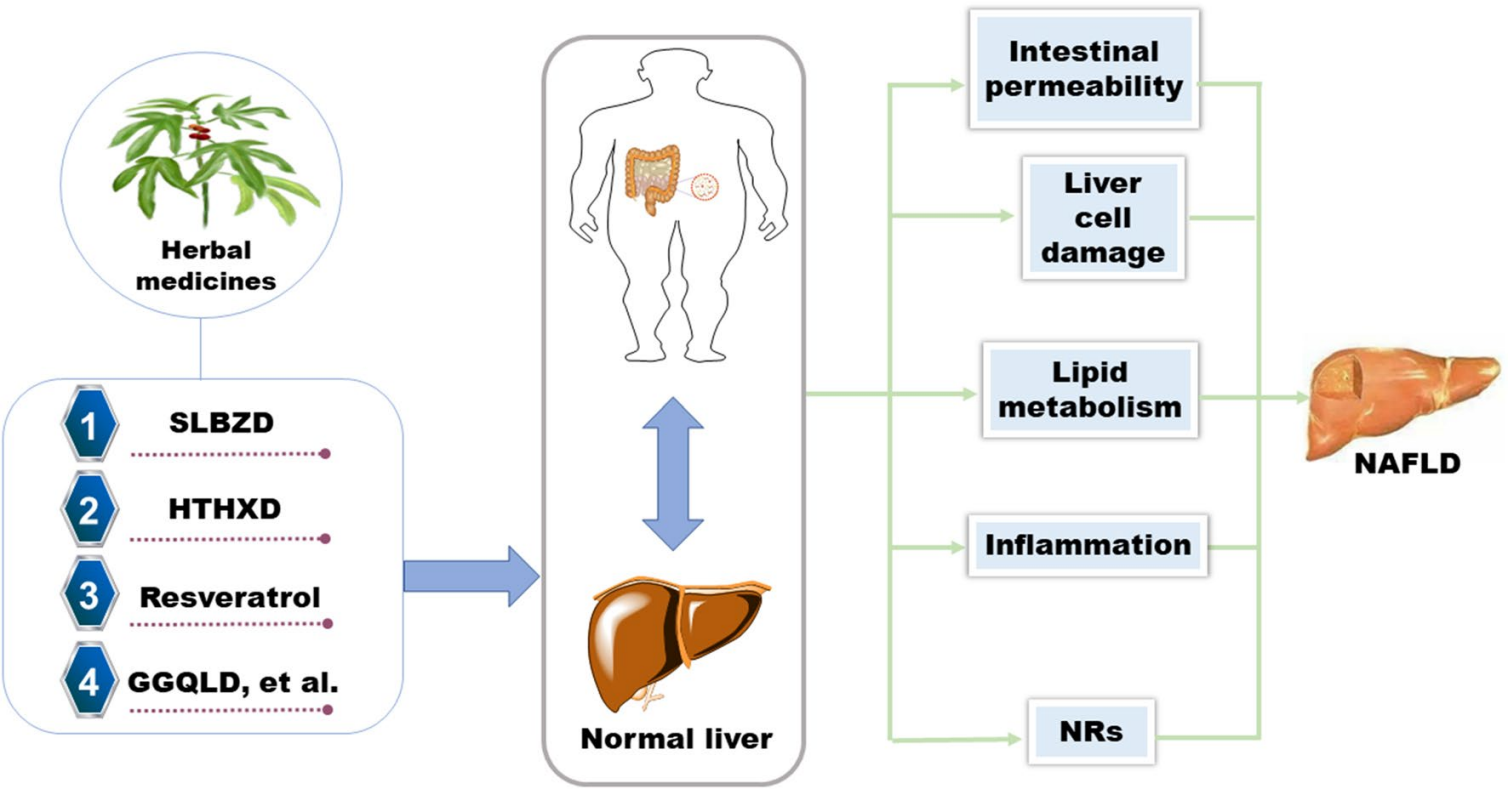
Herbal medicines ameliorate NAFLD via gut microbiota. Some Chinese herbal medicines can activate autophagy through different pathways (Sirt1, Lpl, Scd1, Ppar-γ, Acc1 etc.), and regulate NAFLD-related pathological processes, including intestinal permeability, liver cell damage, lipid metabolism, inflammation and NRs

Unlike Western drugs, herbal medicines are very complex mixtures, and these complex mixtures (as well as their metabolites *in vivo*) may interact with a variety of therapeutic targets in extremely complex ways [116, 117]. In this case, it is necessary to depict the key constituents responsible for the pharmacological effects in humans. Fortunately, most herbal medicines have been validated for a long time and show satisfying safety profiles in humans at recommended dosages. Therefore, more clinical trials of some famous herbal medicines for the treatment of NAFLD should be carefully investigated or reinvestigated, which may provide more powerful evidence from humans and gain new insights into the regulatory mechanism of gut microbiota by herbal constituents. In the future, the long-term use of herbal medicines or the combined use of herbal medicines with Western medicines should also be considered in the clinic, which will be very helpful for the development of new herbal remedies or novel medicines to ameliorate NAFLD by rebuilding intestinal microecology (Fig. 6).

## Data Availability

All data included in this article are available from the corresponding author upon request.

## Abbreviations

NAFLD: Nonalcoholic fatty liver disease
NASH: Nonalcoholic steatohepatitis
HM: Herbal medicines
LPS: Lipopolysaccharide
GGQLD: Gegen Qinlian Decoction
CHMs: Chinese herbal medicines
HFD: High-fat diet
SLBZP: Shenling Baizhu powder
TC: Total cholesterol
TNF-α: Tumour necrosis factor α
IL-β: Interleukin-1β
RES: Resveratrol
PRE: Puerariae radix
PTE: Pu 'er ripe tea
sIgA: Secretory immunoglobulin A
SOD: Superoxide dismutase
MLCK: Myosin light chain kinase
MP-A: Mussel polysaccharideα-D-glucan
FA: Ferulic acid
NRs: Nuclear receptors
PPARα: Peroxisomal proliferator activated receptor α
VDR: Vitamin D receptor
CAR: Constitutive androstane receptor
FXR: Farnesoid X receptor
RORγt: RAR-related orphan receptor γ2
IR: Insulin resistance
HDL-C: High-density lipoprotein cholesterol
LDL-C: Low-density lipoprotein cholesterol
DNL: De novo lipogenesis
BBR: Berberine
FGT: Fermented green tea
